# My preferred approach to left bundle branch pacing: Lumenless leads

**DOI:** 10.1016/j.hroo.2022.12.012

**Published:** 2022-12-27

**Authors:** Shunmuga Sundaram Ponnusamy, Pugazhendhi Vijayaraman

**Affiliations:** ∗ Division of Cardiology, Velammal Medical College, Madurai, India; † Geisinger Heart Institute, Wilkes-Barre, Pennsylvania

**Keywords:** Left bundle branch pacing, His bundle pacing, Lumenless lead, Stylet-driven lead, Left bundle branch block, Cardiomyopathy, Heart failure, Septal perforation

**Key Findings**

▪ Left bundle branch pacing has been increasingly adopted as an excellent strategy of physiological pacing as it provides stable pacing parameters with shorter learning curve
▪ The 3830 bipolar pacing lead is a Food and Drug Administration–approved 4.1F-sized lumenless lead (LLL) with fixed helix and isodiametric tip with excellent tensile strength for performing left bundle branch pacing.
▪ LLLs are associated with minimal myocardial injury, better long-term outcomes, and less incidence of helix damage and procedural complications.
▪ Stylet-driven leads have been recently adopted for left bundle branch pacing, but the experience is limited and currently not approved by Food and Drug Administration.
▪ Globally, LLLs are the most commonly used lead for performing LBBP, as early experience favors LLLs over stylet-driven leads.

## Introduction

Right ventricular (RV) pacing results in electromechanical dyssynchrony in the ventricles leading to increased risk for pacing induced cardiomyopathy, heart failure, atrial fibrillation, and mortality. Physiologic pacing using His bundle pacing (HBP) or left bundle branch pacing (LBBP) maintains ventricular synchrony and in observational, case-control studies has been shown to reduce the risk for death, heart failure hospitalization, or need for upgrade to biventricular pacing when compared with RV pacing.^1^, ^2^, ^3^ HBP has been associated with variable success, a longer learning curve, higher capture thresholds, and increased risk for lead revision. LBBP has gained significant momentum in the recent years due to a shorter learning curve and stable pacing parameters as compared with HBP.^3^, ^4^, ^5^ There are several published reports of LBBP utilizing a thin, SelectSecure magnetic resonance imaging surescan 3830 lead (Medtronic Inc, Minneapolis, MN) with high success rates and low complications. This article describes our approach to LBBP utilizing the 3830 pacing lead.

## Implantation tools

LBBP involves deploying the lead in the basal, muscular interventricular-septum distal to the His bundle. The 3830 bipolar pacing lead is 4.1F in diameter with an inner-cable design and excellent tensile strength and needs to be delivered via special catheters due to its floppy nature. Leads are available at 59-, 69-, and 74-cm lengths. The lead has 2 electrodes at the lead tip made of titanium nitride coated platinum alloy, MP35N nickel alloy conductors, silicone inner insulation, and polyurethane outer insulation. The helix (cathode) length is 1.8 mm with interelectrode distance of 9 mm and can be visualized on fluoroscopy. The helix is coated with beclomethasone dipropionate at target dose of 17.2 μg.

**Figure 1:**
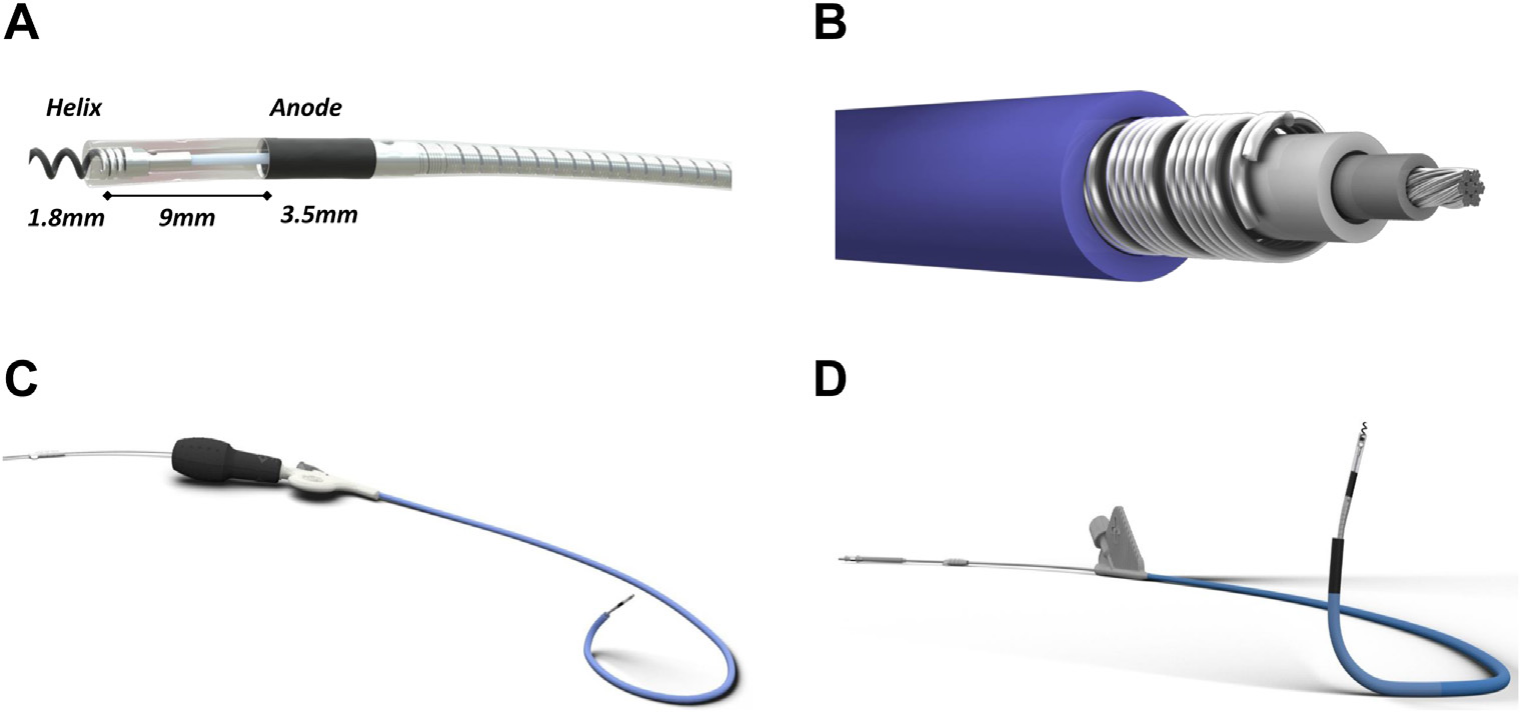
Lumenless lead and delivery sheaths. **A:** The lumenless lead has an open helix of 1.8 mm length with interelectrode distance of 9 mm. **B:** The lumenless lead has an inner conductor surrounded by insulators and coiled outer conductor. **C:** The C304 His sheath is a deflectable one with outer diameter of 8.4F and can be helpful in difficult cases. **D:** The C315 His sheath has fixed 2 curvatures for facilitating entry into the right ventricle and facing toward the septum. Reprinted with permission from Medtronic.

The delivery catheter most used is the preshaped, fixed-curve C315His (Medtronic Inc) sheath with a length of 43 cm, inner diameter of 5.5F, and outer diameter of 7.0F (Figure 1). This sheath has a primary curve to reach the RV and a secondary curve that helps direct toward the septum. A deflectable sheath (C304His; Medtronic Inc) with a septal curve is also available with deflection in one direction (Figure 1); it has an inner diameter of 5.7F and an outer diameter of 8.4F and can be helpful in mapping the septum and in difficult cases. The 3830 lead has received Food and Drug Administration approval for both HBP and LBBP. Several other commercially available delivery sheaths and stylet-driven leads listed in Table 1 have been used for LBBP, but the experience is limited and currently not approved for this purpose by the FDA.

**Table 1:** Currently available tools that can be used for left bundle branch pacing

Delivery catheter	Pacing lead
**Fixed Curve** C315 His (Medtronic) Selectra 3D (Biotronik) SSP (Boston Scientific)	**Lumenless lead** 3830 SelectSecure
**Deflectable** C304 SelectSite (Medtronic) Agilis HisPro (Abbott)	**Stylet-driven leads** Solia S (Biotronik) Ingevity (Boston Scientific) Fineline II Sterox EZ (Boston Scientific) Tendril 2088TC (Abbott)

## Implantation technique

### Lead deployment

Huang and colleagues^6^ first reported the technique of LBBP using the 3830 lead in a patient with LBB block. LBBP is performed by deploying the lead deep inside the proximal interventricular septum 1 to 2 cm from the distal His bundle toward the RV apex.^7^ As it is a lumenless lead (LLL), the body of the lead must be rapidly rotated to drive it inside the septum. It is preferrable that both the lead and the gloves be dry (free of blood, fluid, or contrast) for achieving effective rapid rotations.

**Figure 2:**
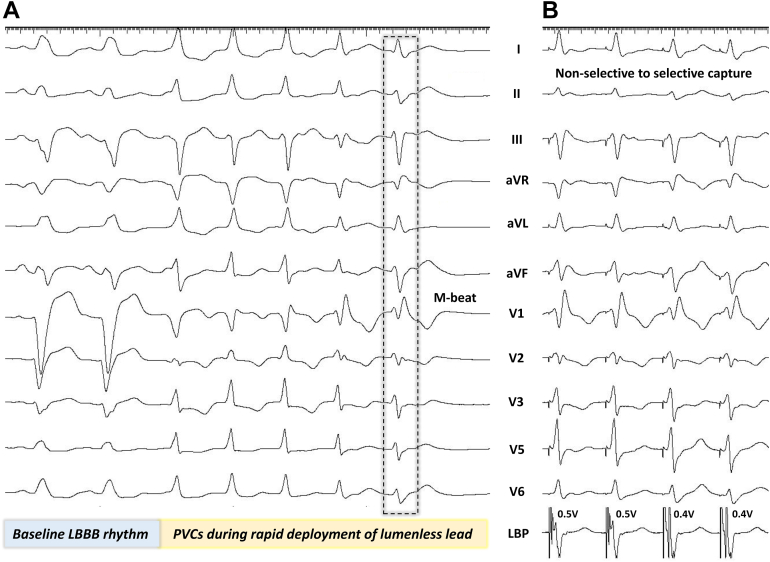
PVC morphology during LBBP. **A:** Premature ventricular complexes during left bundle branch pacing with lumenless lead. Note the change in premature ventricular complex (PVC) morphology from QS to qR as the lead traverse from right to the left side of the septum. The last generated PVC labeled as M-beat had an M pattern in lead V1 with a slurred S-wave in lead V6. The morphology of the M-beat mimicked a selective left bundle branch captured beat. **B:** Nonselective-to-selective capture transition noted at a near-threshold output value. LBBB = left bundle branch block.

The target site for the lead deployment is identified by pacing the proximal septum 1 to 2 cm beyond the distal His bundle where the paced complexes demonstrate a “W” pattern in lead V1 along with discordant complexes in leads aVR and aVL. It is preferred that lead II demonstrates an R or Rs pattern and that leads III and aVF demonstrate an Rs, RS, or rS pattern. Tall R waves in II, III, and aVF would suggest an outflow tract location near the right bundle and should be avoided to prevent injury to the right bundle or large arterial septal perforator branches. Gradual deployment of the lead would result in the notch of the paced QRS in lead V1 to ascend up to form an R-wave along with gradual increase in unipolar pacing impedance. A right bundle branch delay (RBBD) pattern (Qr, qR, QR, or rSR′ in lead V1) during unipolar pacing associated with a sudden drop in unipolar impedance by 100 to 200 Ω would suggest that the lead has reached near the left septal endocardium. Alternatively, the LLL could be deployed by rapid rotations (Video 1), which would generate premature ventricular complexes (PVCs).^8^ The morphology of PVCs would change from QS in lead V1 to qR or rSR in lead V1 as the lead traverses from the right to the left side of the interventricular septum (Figure 2). A PVC with an RBBD pattern labeled as a template or fixation beat^9^^,^^10^ would predict LBB area capture with sensitivity of 96.4% and specificity of 97.3%. With a lumenless pacing lead, template or fixation beats could be observed in 90.5% of the patients.^11^ Template or fixation beats were associated with reduction in fluoroscopy duration and myocardial injury. M-beat (Figure 2), a subset of template beat, had a specificity of 96.7% and sensitivity of 58.6% for predicting selective capture of the LBB.^11^ LBB capture could be confirmed by the presence of RBBD pattern in the paced QRS complex along with any 1 of the following parameters^12^: (1) demonstration of short and constant R-wave peak time in lead V6; (2) demonstration of LBB potential; (3) demonstration of nonselective to selective or nonselective to septal capture transition at near threshold output; (4) V6 to V1 interpeak interval >44 ms; (5) physiology-based electrocardiography criteria; and (6) programmed deep septal stimulation to show change in QRS morphology, axis, and duration.

Stylet-driven leads (SDLs) would require predeployment of the helix before septal positioning is attempted. During attempts at deep septal lead placement, the extendable screw of the SDL can spontaneously retract back, resulting in increase in threshold and pacing impedance. Occasionally, even after successful lead placement at the left ventricular (LV) subendocardium, the screw may retract and needs to be carefully monitored. The distal tip of the LLL is isodiametric and favors easier penetration compared with the nonisodiametric nature of SDL tip.^13^ The SDL may provide additional stiffness due to the stylet and facilitate easier penetration. Continuous pacing can be performed by connecting the pacing cable to the stylet during lead advancement using SDL, which may be an advantage compared with the LLL. Continuous pacing during deployment can also be performed with LLL but requires a special revolving connector pin.^14^

### Septal perforation

**Figure 3:**
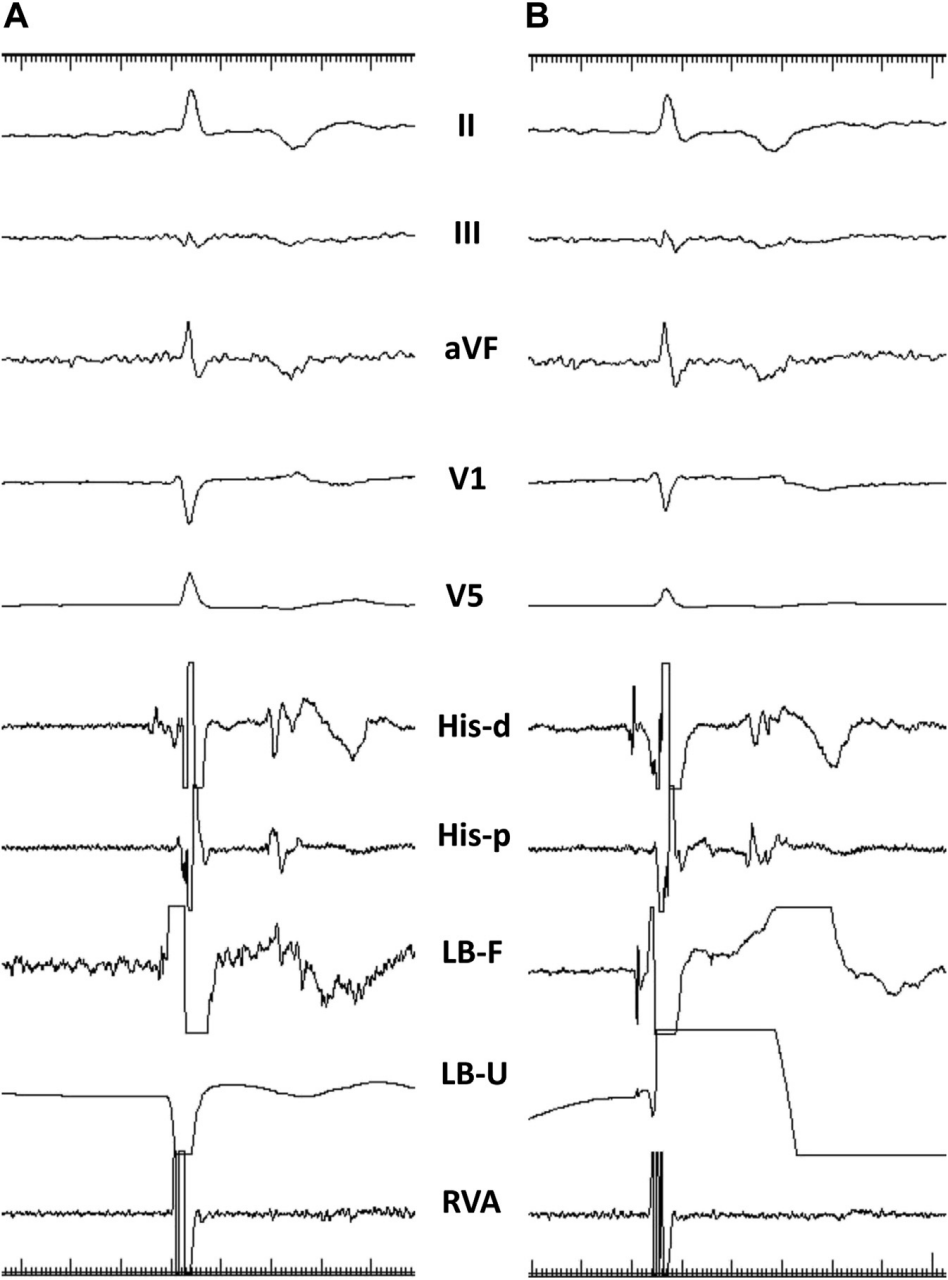
Septal perforation during left bundle branch pacing with lumenless lead. **A:** Note the absent current of injury along with QS pattern in the unfiltered unipolar electrogram (LB-U) during complete perforation into the left ventricular cavity. **B:** After repositioning the same lumenless lead at a different site, an unfiltered electrogram showed significant current of injury along with sharp left bundle potential. His-d = His distal; His-p = His proximal; LB-F = left bundle filtered; LB-U = left bundle unfiltered; RVA = right ventricle apex.

Septal perforation is an important complication to recognize during implantation. The reported acute incidence during implantation varied between 3.2% and 14.1%.^3^^,^^15^^,^^16^ Septal perforation would be benign if recognized promptly and the lead repositioned immediately. Septal perforation into the LV cavity with LLLs have been extensively studied^16^, ^17^, ^18^ and several parameters suggested for easy recognition. We demonstrated septal perforation in 30 (14.1%) of 212 patients who underwent successful LBBP with repositioning of the LLL without significant complications.^16^ A unipolar pacing impedance of <450 Ω (sensitivity 100% and specificity 96.6%) and sudden drop in current of injury (COI) identified septal perforation (Figure 3). LLLs tip connected in unipolar configuration with high- and low-pass filter settings of 0.5 and 500 Hz, respectively, to obtain an unfiltered electrogram would help in monitoring the COI. An unfiltered unipolar electrogram identified 2 different patterns during perforation: (1) type I (QS pattern) in 67% due to complete perforation of the LLL into the LV cavity and (2) type II (RS pattern) in 33% due to partial perforation of the lead tip in the LV cavity.

Shali and colleagues^17^ showed that the absence of COI at the lead tip would indicate acute septal perforation, and the ratio of COI between the tip and ring would indicate the depth of the lead inside the septum, where a very low ratio would suggest microperforation during implantation. Similarly, Orlov and colleagues^18^ showed that the LLL ring impedance showed a stepwise increase during successful attempts as opposed to unsuccessful ones. Ring pacing parameters were more predictive of lead progress inside the septum than the tip parameters and could obviate the need for contrast injection to delineate the lead depth.

The surface area of septal perforation with LLL is small and appears to close with no demonstrable shunting during postprocedure echocardiography.^16^ It is likely that the SDL may result in larger perforated area and may be associated with increased risk for shunts or cameral fistulas^19^^,^^20^ and has not been studied adequately so far.

### Lead repositioning

During LBBP lead implantation, the lead may have to be repositioned at multiple sites due to inability to penetrate or inability to demonstrate LBB capture, or due to perforation into the LV cavity. In a study in which the myocyte injury during LBBP was assessed,^21^ the mean number of attempts for successful lead positioning was 2.5 ± 1.9. Though the troponin release was more in patients requiring >2 attempts as compared with those requiring ≤2 attempts (115.1 ± 85.8 pg/mL vs 71.2 ± 56.1 pg/mL; *P* = .004), there was no difference in outcome during follow-up. In another study in which M-beat as a marker of selective LBB capture was analyzed, both groups of patients with and without an M-beat required a mean of >2 attempts (2.2 ± 1.04 and 2.8 ± 1.09, respectively) for successful lead deployment.^11^ Myocardial injuries during these procedures using LLLs were minimal, and no adverse clinical outcomes were observed. Clinical data regarding myocardial injury and troponin release with SDLs are currently lacking.

Gentle counterclockwise rotation and traction would be sufficient to retract the LLL back from the septum due to its excellent tensile strength. In our study, all 30 patients underwent successful retraction without damaging the helix.^16^ Subsequent repositioning of the LLL was done at a different site with the same lead. Follow-up echocardiography showed no residual ventricular septal defect, shunts, or fistulas, and the pacing parameters remained stable during mean follow-up of 9.9 ± 6.7 months. While repositioning the lead, care must be taken to avoid deploying the lead at the same site by (1) fluoroscopic landmark, (2) pace mapping to show different paced QRS morphology axis on the right side of the septum, (3) demonstrating fixation or template beats, and (4) movement of the lead inside the septum in left anterior oblique fluoroscopic view. Absent template or fixation beats, similar paced QRS axis, and hypertransmission of rotations to the lead tip would indicate same site entry.

Pooter and colleagues^19^ reported 2% (n = 8) incidence of septal perforation during LBBP with SDLs. Though the incidence was less, severe helix damage occurred in 3 of 8 patients, resulting in lead disuse, and one helix fractured inside the septum and could not be retrieved. There was 50% failure in repositioning with the same lead at a different site. SDLs have been reported to be associated with fracture of the helix rotating mechanism and failure to fully extract the pacing lead.^22^ In another study of 280 patients using both LLLs and SDLs for conduction system pacing, SDLs were associated with 29% lead failure (structural damage to the lead necessitating lead replacement) compared with 2% with LLLs.^23^

## Follow-up

### Loss of conduction system capture

LBBP as opposed to HBP has been shown to be associated with low and stable capture threshold during follow-up.^3^, ^4^, ^5^^,^^15^ Loss of LBB capture during follow-up is defined as loss of RBBD (R-wave in lead V1) along with inability to show capture transition (nonselective to selective/septal) during threshold assessment in whom it was documented at the time of implantation. Long-term data on lead performance are available for LLLs as compared with SDLs. Su and colleagues^15^ showed 97.8% acute procedural success rate (618 of 632 patients) with lumenless pacing lead. Pacing parameters remained stable with only 2 patients demonstrating complete loss of conduction system capture (LOCC) during mean follow-up of 18.6 ± 6.7 months. In another 6 patients, LBB capture threshold was >3 V or 0.5 ms with low septal capture threshold. In another study,^24^ LOCC was observed in 15 (4.6%) of 323 patients who underwent successful LBBP with LLL (60% at >6 weeks). A redo procedure was required in 1.5% (n = 5), and the remaining patients had acceptable RV septal capture threshold of <1 V. LOCC was associated with a dilated LV, low LV ejection fraction, and lack of nonselective-to-selective capture transition at implantation. Greater than 3 attempts for successful lead deployment had an odds ratio of 7.55 for LOCC during follow-up. However, SDLs have been shown to be associated with higher incidence of LOCC during follow-up. Tan and colleagues^25^ showed 12% (n = 22) incidence of LOCC among 191 successfully implanted patients during mean follow-up of 230 ± 144 days. LOCC was more frequently observed in patients who received SDLs as compared with LLLs. Lead design was the only independent predictor for LOCC, with 3-fold increased risk for SDLs compared with LLLs. The mechanisms for the higher incidence could be due to different tensile forces during implantation and lack of an effective fixation mechanism during LBBP. With both types of leads, the extended or exposed screw cores the myocardial tissue and simply sits in a “myocardial tunnel” held by the tissue surrounding the length of the lead inside the ventricular septum, unlike the active fixation mechanism of the leads in the RV or right atrial wall, where the lead is anchored by the screw itself. Nonetheless the potential for LOCC appear to be greater with SDL compared with LLL.

### Lead extraction

**Figure 4:**
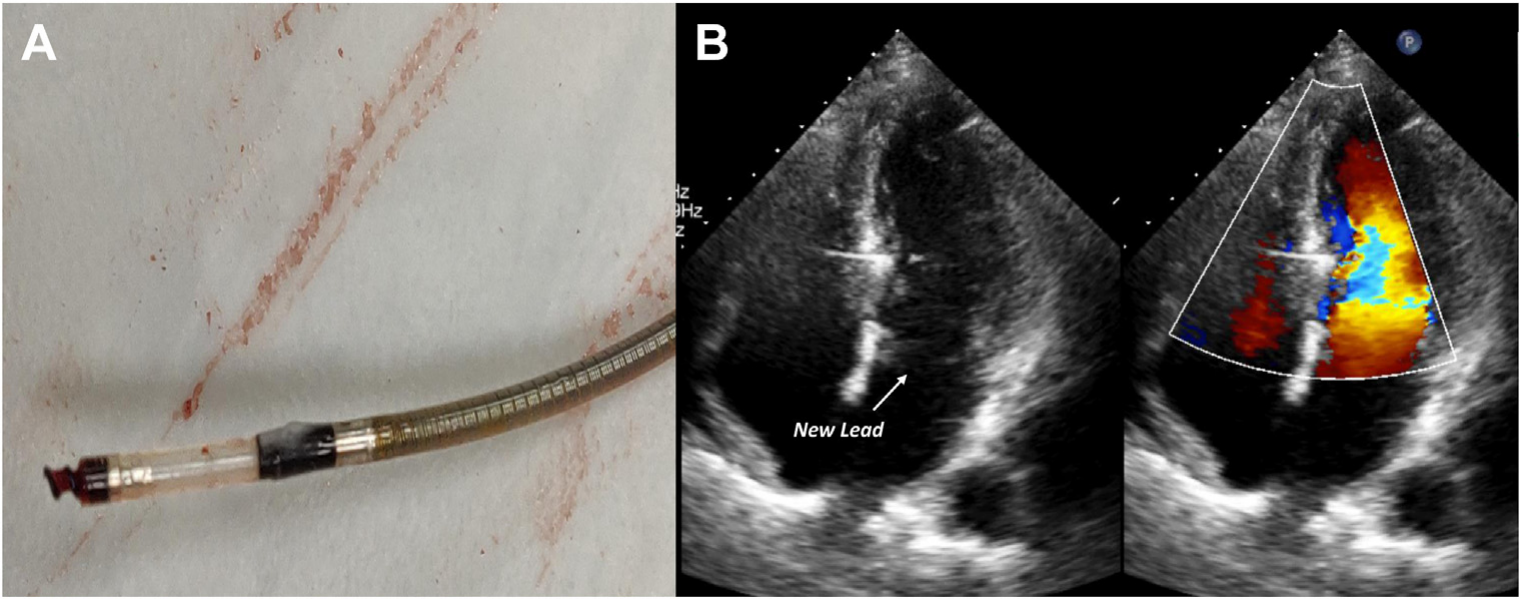
Lumenless pacing lead extraction. **A:** Lead extracted after 2 years en masse without damaging the helix. **B:** Echocardiography showing no significant septal defect after extraction of 1.4-mm sized lumenless lead. Note the position of the new lumenless lead at the midseptum reaching left ventricular subendocardium.

One of the major limitations of LBBP is the paucity of data on lead extraction. The potential risk for extensive fibrosis around the length of the leads (10–20 mm) inside the interventricular septum and the risks associated with attempted extraction from this location following long dwell times are currently unknown. There are very few reports of lead extraction from the LBBP location,^26^^,^^27^ with the longest duration of 3 years (unpublished personal observation). Surprisingly, the LLLs were easily extracted with simple manual traction in all these cases (Figure 4 and Video 2). There are no data on extraction of SDLs from the LBB area, as these leads were only recently adopted for LBBP. The LLL may have a potential advantage due to its inner-cable design, which acts as an excellent locking stylet for lead extraction providing significant tensile strength, as has been demonstrated during extraction of the 3830 leads from the HBP location^28^ and other sites.^29^

### Procedural complications

Apart from septal perforation which require immediate recognition and repositioning, septal hematoma,^30^ coronary artery injury,^31^ septal coronary artery fistula,^19^ and lead dislodgements are the reported complications of LBBP. Though theoretically possible, so far thromboembolic complications have not been reported both with both LLLs and SDLs after LBBP. Septal coronary-cameral fistula been reported with SDLs probably due to the larger lead dimension compared with LLLs. Recently, a case report of fracture of an SDL a few months after implantation was reported.^32^ Long-term follow-up of these leads at the LBBP location is necessary to assess the integrity of these leads due to extraordinary rotational stress on the leads during implantation and subsequent impact of repeated contractile force of the septal myocardium especially between the ring and tip electrodes.

### Why would I choose LLL for LBBP?

Table 2 shows the comparison of LLLs and SDLs for LBBP. A small caliber pacing lead with isodiametric tip would help in improving the procedural success by facilitating better penetration into the interventricular septum with minimal myocyte injury. Helix retraction during follow-up resulting in loss of conduction system capture would be avoided, as LLLs have a fixed helix at the tip. Multiple attempts might be required in some patients before successful lead deployment and LLLs have the advantage over SDLs, as they are associated with less incidence of helix fracture. Extraction of LLLs if required would be feasible due to their high tensile strength, as demonstrated in few case reports, though long-term studies are required to demonstrate the feasibility of extracting LLL with long dwelling period. Finally, LLLs are well supported by long-term studies demonstrating their safety and efficacy for performing LBBP, which is lacking with SDLs.

**Table 2:** Comparison of lumenless and stylet-driven leads for left bundle branch pacing

Parameter	Lumenless lead	Stylet-driven lead
Lead size, F	4.1	5.6/5.7/5.8
Lead length, cm	59/69/74	53–85
Helix length, mm	1.8	1.8
Interelectrode distance, mm	9	10/10.7
Isodiametric tip	Yes	No
Steroid at lead tip	Beclomethasone	Dexamethasone
Potential for helix damage	Less likely	More likely
Long-term studies	Available	Nil
Potential for myocardial injury	Less	More
Lead repositioning	Possible	difficult
Risk of LBB capture loss	Less	More
Lead extraction	Feasible	?
Septal coronary-cameral fistula	Rare	More likely

LBB = left bundle branch.

## Future directions

LBBP is an acceptable alternative strategy for both RV and biventricular pacing, as it provides stable pacing parameters and corrects distal conduction system disease. Septal scar is considered as one of the major limiting factor for procedural success. Improvisation in the lead design and delivery tools are necessary to increase the ease and success rates of LBBP lead implantation in patients with ischemic heart disease, septal scar, or dilated atria or ventricles. Modification to the fixation mechanism may be necessary to prevent loss of conduction system capture. The long-term risks of lead extraction in patients undergoing LBBP need to be carefully evaluated in future studies. The feasibility and success rates of LBBP utilizing SDLs in patients requiring cardiac resynchronization therapy have not been well studied. Randomized studies are necessary to better assess the advantages and disadvantages of LLLs vs SDLs for LBBP in various patient populations.

## Conclusion

LBBP is a recent innovation in the pacing strategy with excellent short- and medium-term pacing outcomes. LLLs have relatively longer follow-up data with larger patient populations compared with SDLs. While continuing to refine the implantation tools and techniques, we currently prefer LLLs over SDLs in patients undergoing LBBP for most pacing indications.
